# Consummatory, Feeding Microstructural, and Metabolic Effects Induced by Limiting Access to Either a High-Sucrose or a High-Fat Diet

**DOI:** 10.3390/nu12061610

**Published:** 2020-05-30

**Authors:** Harrison Sunjoon Lee, Elisa Giunti, Valentina Sabino, Pietro Cottone

**Affiliations:** Laboratory of Addictive Disorders, Departments of Pharmacology and Psychiatry, Boston University School of Medicine, Boston, MA 02118, USA; hsl9@bu.edu (H.S.L.); egiunti@bu.edu (E.G.); vsabino@bu.edu (V.S.)

**Keywords:** binge eating disorder, palatable diet, microstructure

## Abstract

Background: Binge eating disorder (BED) is characterized by recurrent binge eating episodes consisting of rapid consumption of excessive amounts of highly palatable, energy-dense food within discrete periods of time. The aim of this study was to test the consummatory, food microstructural, and metabolic effects of a one hour limited access to either a high-sucrose diet (HSD) or a high-fat diet (HFD) in an operant rat model of binge-like eating. Methods: Female rats were subject to a binge-like eating procedure in which a HSD, a HFD, or a standard chow diet were provided in a fixed ratio 1 (FR1) operant schedule of reinforcement. Results: Limiting access to either a HSD or a HFD promoted binge-like eating as compared to the control chow diet. However, binge-like eating of HSD, but not HFD, was based on a true increase in the amount of food consumed, an increased eating rate, and a decrease in the intake of the home-cage standard chow, altogether suggesting an increase in palatability. Moreover, while HSD rats consumed overall less energy than HFD rats, the former were more energy efficient and gained more body weight than the latter. Conclusions: These results provide information on how the quality of food can deeply influence the behavioral and metabolic outcomes of binge-like eating.

## 1. Introduction

Binge eating disorder (BED) is an eating disorder characterized by discrete and recurrent episodes of binge eating, which involves the rapid consumption of excessive amounts of food within a discrete time period, accompanied by a sense of loss of control [1,2,3,4]. The lifetime prevalence of BED in the United States is estimated at 2.6% and, similar to other eating disorders, it occurs more commonly in women than men [5]. A large proportion of people suffering from BED also have other psychiatric disorders and medical comorbidities [1,6]. BED was first coded as a separate eating disorder in the fifth edition of the *Diagnostic and Statistical Manual of Mental Disorders* (DSM-5) [1].

In preclinical research, a common experimental procedure used in the development of animal models of binge-like eating consists of exposing the subjects to either sugar-rich or fat-rich food for a limited period of time [7,8,9,10]. The rationale for using such an expedient is twofold: as discussed earlier, binge episodes occur in brief periods of time, and also, the food ingested by people suffering from BED is typically energy-dense and highly palatable [11].

While the majority of the existing animal models of binge-like eating are performed using home-cage experimental feeding procedures, our laboratory has developed, validated, and characterized a rat model of binge-like eating which is based on daily limited access to a palatable diet through an operant conditioning procedure [12,13,14,15,16,17,18]. In this operant model of binge-like eating, rats are allowed to self-administer, via a nose-poke, food pellets of either a standard chow diet or a high sucrose diet in 1-h daily sessions [16,18]. In addition to the intrinsic features of instrumental conditioning, the use of an operant procedure offers two critical advantages over a traditional measurement of food weight: (i) food intake is measured by the number of responses for very small units of food (45 mg) and, therefore, feeding results are from the contribution of hundreds of small events rather than a single food weight measurement; and (ii) measurements are taken at a very high temporal resolution (10 msec), allowing for a very detailed analysis of food responding over time. These two properties have been used to give a fine microstructural description of the rate and regularity of intake through the analysis of the intervals between consecutive feeding responses, which otherwise would not be possible with traditional measurements of food. Such microstructural analyses acquire a particular relevance in the context of animal models of BED as an increased eating rate is a criterion used for the diagnosis of the disorder [13,18].

While our laboratory has published a number of studies to understand the effects of a high-sucrose diet on the operant model of binge-like eating [12,13,14,15,16,17,18], the effects of a high-fat diet in such a model are unknown.

Therefore, the aim of this study was to compare the consummatory, the feeding microstructural, and the metabolic effects of limiting access to either a high-sucrose or a high-fat diet in female rats.

## 2. Materials and Methods

### 2.1. Subjects

Female Sprague Dawley rats (*n* = 30) (Charles River, Wilmington, MA, USA), 45 days old and weighing 170–220 g upon arrival, were singly housed in wire-topped plastic cages (27 × 48 × 20 cm). Rats were housed in a 12/12 h reverse light cycle (lights off at 11 am) in a humidity- and temperature-controlled vivarium. Rats had access to corn-based chow (Harlan LM-485 Diet 7012 (65% (kcal) carbohydrate, 13% fat, 21% protein, 3.1 kcal/g); Harlan, Indianapolis, IN, USA) and water ad libitum in their home cage. All experimental procedures adhered to the National Institute of Health Guide for the Care and Use of Laboratory Animals and were approved by the Boston University Institutional Animal Care and Use Committee (IACUC—PROTO201900098).

### 2.2. Apparatus for Self-Administration Procedures

The self-administration procedure was conducted in operant test chambers (30 × 24 × 29 cm) (Med Associates Inc., St. Albans, VT, USA). Each chamber had a stainless steel grid floor and was located inside a ventilated sound-attenuating enclosure (66 × 56 × 36 cm) [18,19]. Pellet dispensers delivered food reinforcers (45 mg pellets) into pellet receptacles, while solenoids delivered water reinforcers (0.1 mL water) into adjacent liquid cup nose-poke receptacles., and 28-V stimulus cue lights were located above each lever and above the food magazine. Light cues (lasting 0.5 s) were paired with the delivery of pellets and water. All responses were recorded automatically by a microcomputer with 10 ms resolution.

### 2.3. Operant Binge-Like Eating Procedure in Ad Libitum Fed Rats

**Figure 1:**
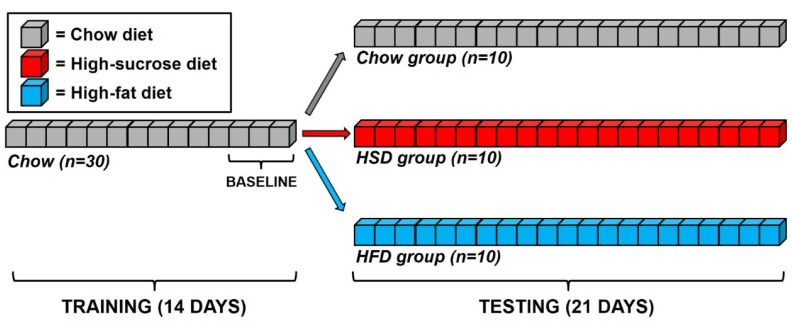
Schematic representation of the timeline of the study. Briefly, rats were trained to nose-poke respond to obtain 45 mg pellets of a standard chow diet under a fixed-ratio 1 (FR1) schedule of reinforcement for 14 days. Upon stabilization of responding, rats were matched and subdivided into three experimental groups: one set (10 rats) was assigned to a chow group (Chow) and received the same 45 mg pellets received in the training phase; a second set (10 rats) was assigned to a high-sucrose diet (HSD) group and received high-sucrose 45 mg pellets; a third set (10 rats) was assigned to a high-fat diet (HFD) group and received high-fat 45 mg pellets. Testing lasted 21 days.

*Training:* One day after arrival, rats were provided an AIN-76A-based diet as a home-cage diet. Hereafter, the AIN-76A-based diet is referred to as “chow” (test diet, 5TUM diet formulated as 4–5 g extruded pellets (65.5% (kcal) carbohydrate, 10.4% fat, 24.1% protein, 3.3 kcal/g); Richmond, IN, see Supplementary Table S1). As previously described [16,18,19], rats were trained to self-administer AIN-76A-based 45 mg precision food pellets (chow) and water (0.1 mL) under a fixed ratio 1 (FR1) schedule of reinforcement in the operant chambers for 14 days (see Figure 1). During these training sessions, the 45 mg pellets had an identical composition to the home-cage diet (chow). Food composition was kept the same in order to ensure that food intake during operant sessions was influenced only by homeostatic factors [18,19]. Daily operant 1-h sessions took place 1–2 h before the dark cycle onset. The reason behind this experimental choice was to naturally increase the drive for food following the sleep cycle in ad libitum fed rats and to obtain robust and highly stable food intake. Intraclass correlation (ICC) analysis of food responding of rats during the last three days of training showed very stable food responding (ICC (2,3) = 0.83; F(29,58) = 5.92, *p* < 0.00000001).

*Testing:* After establishing stable baseline performance in the daily 1-h FR1 sessions, rats were matched for their body weight, daily food intake, feed efficiency, and water and food responding in the sessions, and they were subdivided into three experimental groups. One set (10 rats) was assigned to a chow group (Chow) and received the same 45 mg pellets received in the training phase. A second set (10 rats) was assigned to a high-sucrose diet (HSD) group and received 45 mg pellets that were composed of a nutritionally complete, chocolate-flavored, high-sucrose AIN-76A-based diet. This sucrose diet was comparable in macronutrient composition and energy density to the chow diet (test diet, chocolate flavored 5TUL diet formulated as 45 mg precision food pellets (66.7% (kcal) carbohydrate, 12.7% fat, 20.6% protein, 3.44 kcal/g); Richmond, IN, Supplementary Table S1); previous experiments have demonstrated that all rats strongly prefer this chocolate-flavored diet [20,21]. A third set (10 rats) was assigned to a high-fat diet (HFD) group and received 45 mg pellets with the following composition: Bio-Serv, custom F07679 diet formulated as 45 mg precision food pellets (25% (kcal) carbohydrate, 60% fat, 15% protein, 5.23 kcal/g; Flemington, NJ, Supplementary Tables S1 and S2). Subjects were tested daily for 21 consecutive days and were never food restricted/deprived.

### 2.4. Rate and Regularity of Sustained Eating: Inter Food Interval Analysis

To identify differences among chow vs. HSD vs. HFD rats in the rate and regularity of sustained eating, analysis of the ln-transformed duration of consecutive (uninterrupted by drinking) inter food intervals were performed [22,23]. The inter food interval is a variable inversely correlated to the eating rate. The mean, skewness, kurtosis, and histogram entropy of the ln-transformed duration of each subject’s consecutive inter food intervals were individually determined and then averaged across subjects. The normalized frequency histogram entropy (*H*) is a measure of categorical variability in the rate of ingestion [24], and it was computed as follows:

$$H=-\frac{\sum_{i}^{n}p_{i}\;\log_{2}\;\left(p_{i}\right)}{\log_{2}\;\left(n\right)}$$

*H* is scaled between 0 and 1, with the denominator determined by the number of possible bins in the histogram (*n*) and the numerator by a function of the proportion of observations that fall within a given histogram bin (*p_i_*). Minimal (*H* = 0) entropy occurs when all observations occur within a single histogram bin, whereas maximal entropy (*H* = 1) occurs when each histogram bin has an equal probability, or a flat uniform joint density distribution. For entropy analysis, histograms were constructed from ln-transformed inter food intervals that fell from *e*^−1^ to *e*^3^ sec (~0.34–20.1 s), with a bin width of *e*^0.1^.

Significant decreases in the mean indicate an increased eating rate. Similar to licking rate, this effect has been operationalized as a hallmark of increased palatability [18,25,26]. Significant increases in the histogram entropy (a measure of categorical variability, reflected in an increasing number of populated histogram bins, each with more similar event frequencies), indicate a decreased regularity of intake. Conversely, a decrease in the kurtosis of the inter food interval distribution (a measure of the distribution’s “peakedness”, reflected in a flatter top and taller tails of the distribution), is consistent with a decreased regularity of pellet-to-pellet feeding. Finally, a significant increase in the skewness (a measure of the distribution’s symmetry, reflected in a selective increase of the frequency of the inter food interval falling to the left of the histogram) is consistent with a selective increase of the fast pellet-to-pellet responding.

*Return map analysis.* Return maps, or joint inter-event interval plots, are a nonlinear method of time series analysis that reveals the serial temporal organization of discrete events in time [22,27,28]. For return map analysis, each inter food interval (IFI) in the time-series was scatter-plotted against its subsequent IFI (IFI+1) in a Cartesian plane. In such analysis, highly regular and rhythmic events are observed as densely focused clusters of points. Joint probability density distributions were constructed from return maps and appeared in a color-coded form with warmer colors (toward red) indicating higher local probabilities in the color figure.

### 2.5. Home-Cage Food Intake and Body Weights

23-h home-cage food intake (g) and body weights (g) of subjects were measured daily before each self-administration session. 23-h home-cage food intake was calculated as the food consumed between the end of a self-administration session and the beginning of the next one. Kcal values were calculated by multiplying the values in grams by the caloric density of the different diets (3.3 kcal/g for chow, 3.44 for HSD, and 5.23 for HFD). Daily 24-h food intake (kcal) was calculated as the sum of the 23-h home-cage food intake (kcal) and 1-h food intake self-administered into the operant chambers (kcal). Cumulative 1-h food intake in g and kcal were calculated as the sum of the individual 1-h food intake in g and kcal respectively throughout the 21 days of study. Cumulative body weight gain at any given day was calculated as the body weight gained between the beginning of the self-administration testing and that certain day. Cumulative food intake (kcal) was calculated as the sum of daily 24-h food intake (kcal). Average and cumulative feed efficiency was calculated as body weight change (mg) gained in a certain period of time divided by the food intake (kcal) in the same period [29].

### 2.6. Statistical Analysis

1-h food intake (g and kcal), 24-h food intake (kcal), 23-h food intake (kcal), cumulative body weight gain (g), IFI (Ln(sec)), entropy, skewness, kurtosis, cumulative food intake (kcal), and feed efficiency (mg/kcal) during the 21 days of self-administration were analyzed using two-way analyses of variance (ANOVAs) with diet as a between-subject factor and day as a within-subject factor. Cumulative 1-h food intake (g), and cumulative 1-h food intake (kcal) were analyzed using one-way ANOVAs with diet as a between-subject factor. A Newman–Keuls test was used to interpret significant group differences on the individual days. Pearson’s coefficients were calculated between the 23-h food intake and 1-h food intake, IFI, or entropy, respectively. For such analyses, the averages of days 13–21 were used, a period in which HSD 23-h food intake started being significantly lower than controls.

The software packages used were SigmaPlot 14.0 (Systat Software, Inc., Point Richmond, CA, USA), Instat 3.0 (GraphPad Software, Inc., San Diego, CA, USA), Origin 8.5 (OriginLab Corporation, Northampton, MA, USA), and Statistica 12.0 (StatSoft, Tulsa, OK, USA).

## 3. Results

### 3.1. Consummatory Effects Induced by Limiting Access to Either a High-Sucrose or a High-Fat Diet

**Figure 2:**
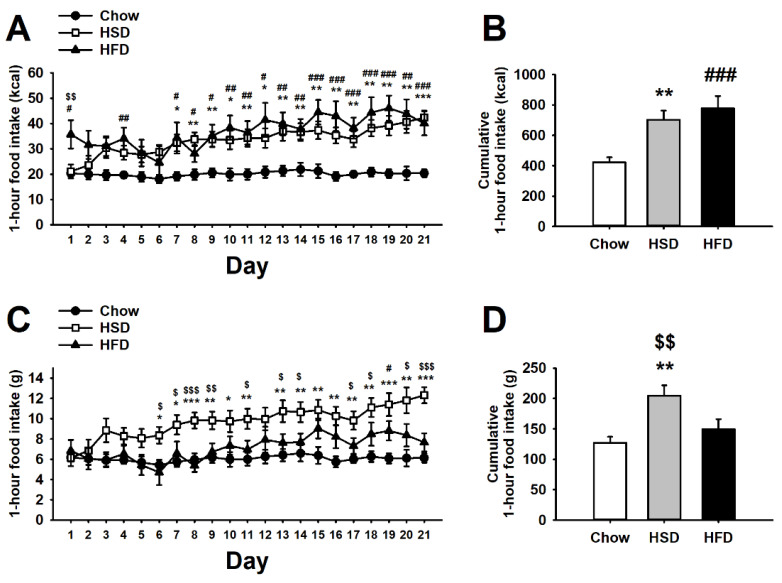
Effects of daily 1-h operant self-administration of Chow, HSD, or HFD on (**A**) food intake (kcal), (**B**) cumulative food intake (kcal), (**C**) food intake (g), and (**D**) cumulative food intake (g) in female Sprague Dawley rats (*n* = 10/group). Rats were given the chance to self-administer one of the three diets in a fixed ratio 1 schedule during a 21-day period. Chow was otherwise freely available in the home cages. Panels represent *M* ± SEM. Symbols denote the following: * HSD differs from Chow, *p* < 0.05, ** *p* < 0.01, *** *p* < 0.001; # HFD differs from Chow, *p* < 0.05, ## *p* < 0.01, ### *p* < 0.001; $ HSD differs from HFD, *p* < 0.05, $$ *p* < 0.01, $$$ *p* < 0.001 (Newman–Keuls test).

As shown in Figure 2A, the statistical analysis of the energy (kcal) of food consumed during the 1-h self-administration sessions revealed a significant main effect of diet (F(2,27) = 9.17, *p* < 0.001), a significant main effect of day (F(20,540) = 7.20, *p* < 0.001), and a significant interaction of diet * day (F(40,540) = 2.33, *p* < 0.001). As expected, while chow rats showed a steady energy consumption throughout the entire period of study, HSD rats instead escalated and doubled their energy consumption as compared to controls. Interestingly, the intake of HFD rats remained steadily higher than Chow rats since day 1 and for most of the observation time window. HFD and HSD rats differed in kcal consumed only at the beginning of the diet exposure, but the difference disappeared quickly. Therefore, both HSD and HFD rats consumed cumulatively more kcal than Chow control rats (diet: F(2,27) = 9.17, *p* < 0.001; Figure 2B).

Interestingly, when the quantity of grams of food, rather than kcal ingested during the 1-h self-administration sessions, was analyzed, it revealed a different perspective on experimental subjects’ feeding behavior (diet: F(2,27) = 7.05, *p* < 0.01; day: F(20,540) = 8.84, *p* < 0.001; diet * day: F(40,540) = 2.85, *p* < 0.001; Figure 2C). Different from the kcal consumed, given the higher energy-density of the high-fat diet as compared to the standard diet (5.23 vs. 3.30 kcal/g, HFD vs. chow diet, respectively), the grams of food ingested by HFD rats did not reliably increase across the 21 days, except for days 15 and 16, as compared to Chow control intake. Conversely, since the high-sucrose diet was similar in caloric density as the control diet (3.44 vs. 3.30 kcal/g, HSD vs. chow diet, respectively), analyses of either grams or kcal of food consumed showed similar patterns of HSD vs. Chow differences throughout the 21 days of the study. Therefore, while HFD binge eating was largely due to the “passive” intake of kcal, HSD binge eating was given by a factually bigger amount of food eaten throughout the study (diet: F(2,27) = 7.05, *p* < 0.01; Figure 2D).

### 3.2. Feeding Microstructural Effects Induced by Limiting Access to Either a High-Sucrose or a High-Fat Diet

**Figure 3:**
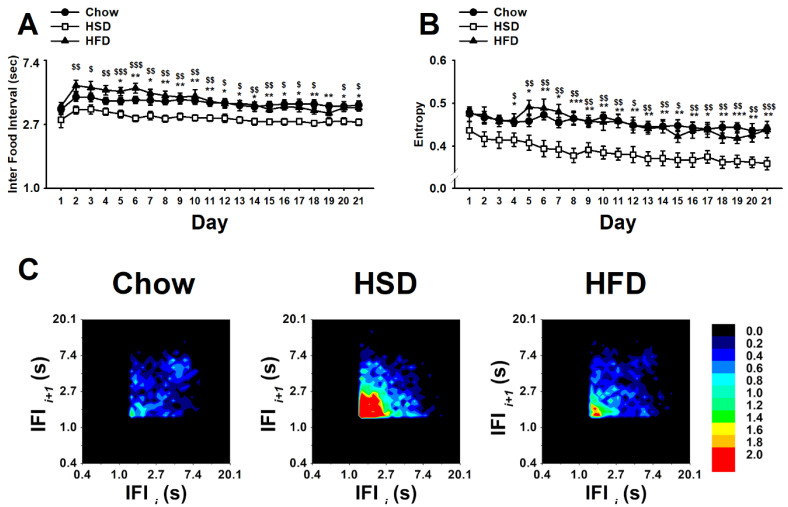
Effects of daily 1-h operant self-administration of Chow, HSD, or HFD on (**A**) inter food interval (sec), (**B**) entropy, and (**C**) return map in female Sprague Dawley rats (*n* = 10/group). Rats were given the chance to self-administer one of the three diets in a fixed ratio 1 schedule during a 21-day period. Chow was otherwise freely available in the home cages. Panels represent *M* ± SEM. Symbols denote the following: * HSD differs from Chow, *p* < 0.05, ** *p* < 0.01, *** *p* < 0.001; $ HSD differs from HFD, *p* < 0.05, $$ *p* < 0.01, $$$ *p* < 0.001 (Newman–Keuls test).

As shown in Figure 3A, the statistical analysis of the ln-transformed duration of consecutive (uninterrupted by drinking) inter food intervals (IFIs) revealed a significant main effect of diet (F(2,27) = 8.67, *p* < 0.01), a significant main effect of day (F(20,540) = 12.42, *p* < 0.001), and a significant interaction diet * day (F(40,540) = 1.66, *p* < 0.01). Chow control rats showed steady IFIs across the 21 days of observation. However, HSD rats showed a reliable decrease in IFIs as compared to Chow controls starting from day 1 and throughout the entirety of the study. Unexpectedly, HFD rats showed an increasing trend as compared to Chow rats during the first week of operant self-administration to then quickly stabilize to a control level. Therefore, the high-sucrose diet, but not the high-fat diet, was able to reliably increase the rate of intake as compared to controls. Surprisingly, while HSD rats showed an increased regularity of sustained eating compared to Chow rats, as revealed by a highly significant decrease in the entropy, the HFD rats’ food responding consistency did not differ from controls (diet: (F(2,27) = 10.13, *p* < 0.001); day: (F(20,540) = 10.26, *p* < 0.001); diet * day: (F(40,540) = 1.25, *n.s.*); Figure 3B). As expected [18], no differences in skewness or kurtosis were observed (skewness, diet: (F(2,27) = 2.79, *n.s.*); day: (F(20,540) = 2.87, *p* < 0.001); diet * day: (F(40,540) = 1.10, *n.s.*); kurtosis, diet: (F(2,27) = 1.55, *n.s.*); day: (F(20,540) = 1.84, *p* < 0.05); diet * day: (F(40,540) = 1.40, *n.s.*); not shown). Accordingly, the return map analysis of the 21st day of testing showed that, compared to the homogeneously distributed IFI values of Chow controls throughout the entire map, food responding of the HSD rat group was characterized by a denser cluster of IFIs skewed in the bottom left of the map (Figure 3C). In agreement with the IFI and entropy results shown above, the HFD rats’ return map was distributed more across a wide range of IFIs and, therefore, resembled more that of Chow rats rather than that of HSD rats.

**Figure 4:**
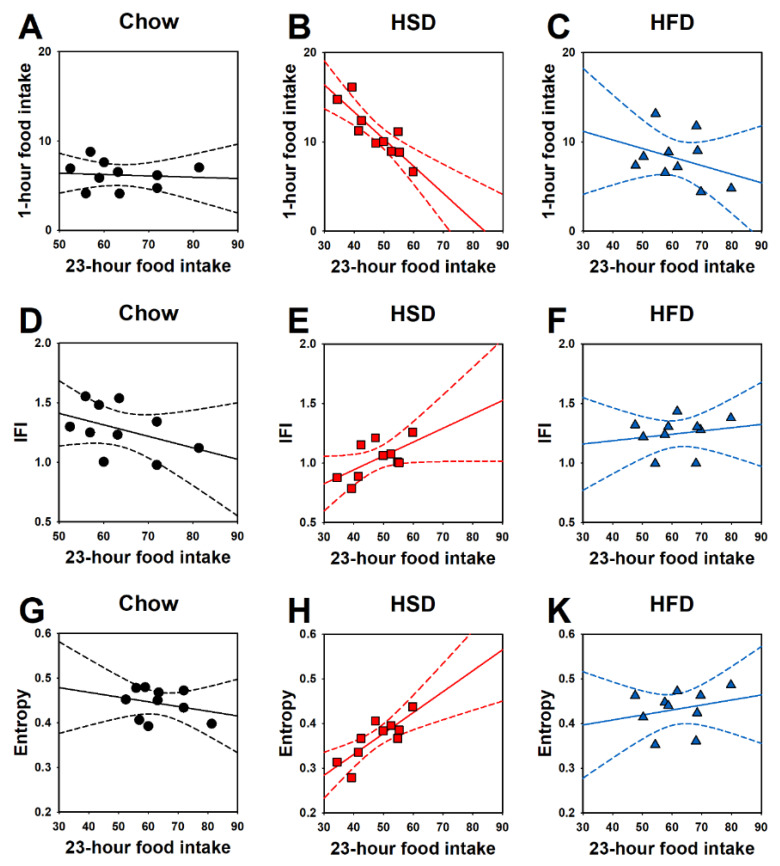
Effects of daily 1-h operant self-administration of Chow, HSD, or HFD on correlations between 23-h home-cage food intake and (**A**–**C**) 1-h food intake, (**D**–**F**) inter food interval (IFI), or (**G**, **H**, and **K**) entropy in female Sprague Dawley rats (*n* = 10/group). For such analyses, the averages of days 13–21 were used, a period in which HSD 23-h food intake started being significantly lower than controls. Rats were given the chance to self-administer one of the three diets in a fixed ratio 1 schedule during a 21-day period. Chow was otherwise freely available in the home cages.

Interestingly, the 23-h calories consumed in the home cages by HSD rats significantly and negatively correlated with the amount of food consumed in the 1-h operant sessions (r(8) = −0.87, *p* < 0.001; Figure 4B), approached a statistically significant positive correlation with IFI (r(8) = 0.62, *p* = 0.054; Figure 4E), and significantly and positively correlated with entropy (r(8) = 0.82, *p* < 0.01; Figure 4H). Such correlations were found in neither Chow nor HFD rats (23-h food intake vs. 1-h food intake in chow rats, r(8) = −0.08, *p* = 0.81; Figure 4A, and in HFD rats, r(8) = −0.35, *p* = 0.33; Figure 4C; 23-h food intake vs. IFI in chow rats, r(8) = −0.42, *p* = 0.23; Figure 4D, and in HFD rats, r(8) = 0.19, *p* = 0.61; Figure 4F; 23-h food intake vs. entropy in chow rats, r(8) = −0.28, *p* = 0.43; Figure 4G, and in HFD rats, r(8) = 0.24, *p* = 0.49; Figure 4K).

### 3.3. Metabolic Effects Induced by Limiting Access to Either a High-Sucrose or a High-Fat Diet

**Figure 5:**
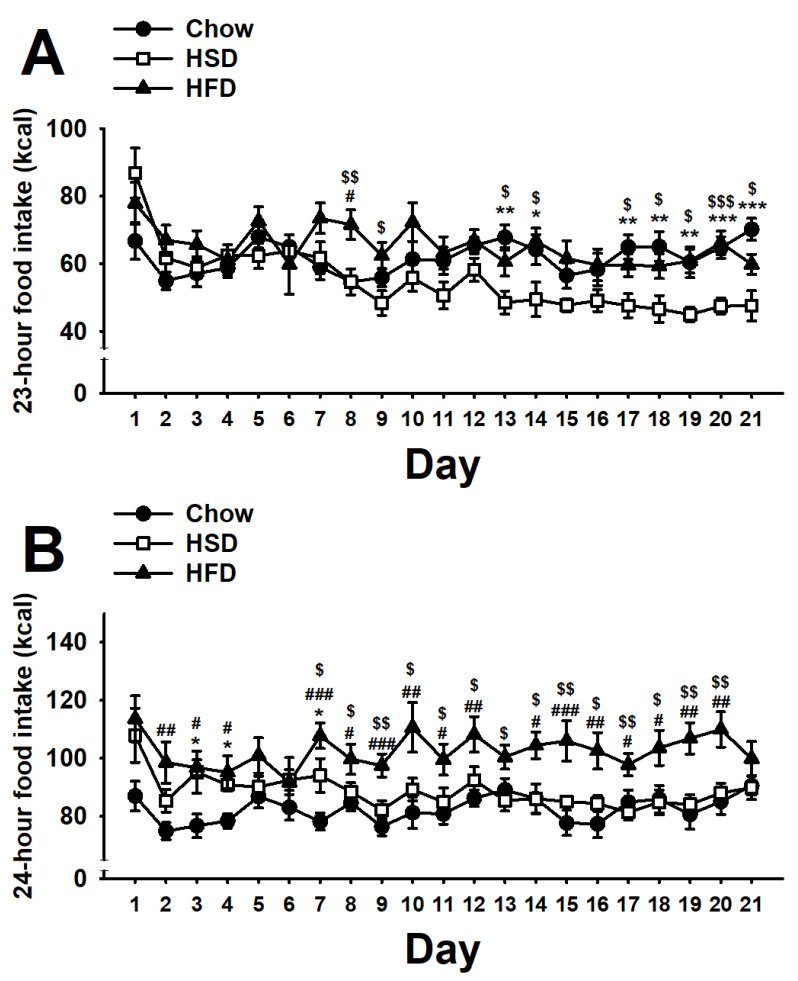
Effects of daily 1-h operant self-administration of Chow, HSD, or HFD on (**A**) 23-h home-cage food intake (kcal), and (**B**) 24-h food intake (kcal) in female Sprague Dawley rats (*n* = 10/group). Rats were given the chance to self-administer one of the three diets in a fixed ratio 1 schedule during a 21-day period. Chow was otherwise freely available in the home cages. Panels represent *M* ± SEM. Symbols denote the following: * HSD differs from Chow, *p* < 0.05, ** *p* < 0.01, *** *p* < 0.001; # HFD differs from Chow, *p* < 0.05, ## *p* < 0.01, ### *p* < 0.001; $ HSD differs from HFD, *p* < 0.05, $$ *p* < 0.01, $$$ *p* < 0.001 (Newman–Keuls test).

As shown in Figure 5A, the statistical analysis of the 23-h home-cage food intake revealed a significant main effect of diet (F(2,27) = 4.50, *p* < 0.05) a significant main effect of day (F(20,540) = 6.22, *p* < 0.001), and a significant interaction of diet * day (F(40,540) = 2.98, *p* < 0.001). Chow rats showed a steady home-cage intake throughout the study. While HFD rats did not reliably show any difference as compared to control Chow rats, HSD rats showed a progressive and slow decrease in the home cage food intake as compared to both Chow and HFD rats.

The statistical analysis of the 24-h daily food intake revealed important differences between HSD and HFD groups during the 21 days of the study (diet: F(2,27) = 11.30, *p* < 0.001; day: F(20,540) = 2.81, *p* < 0.001; diet * day: F(40,540) = 1.42, *p* < 0.05; Figure 5B). Consistent with the steady 1-h self-administration and 23-h home-cage food intake, chow rats showed a stable daily food intake throughout the 21-day observation period. HSD rats consumed more energy than controls during the 1-h operant self-administration session, but decreased their food intake in the home cage, and therefore did not reliably differ from Chow control rats on most days. Conversely, HFD rats, which consumed more energy than controls during the 1-h operant self-administration session, but did not decrease their 23-h home-cage food intake, showed a reliable increase in the 24-h food intake as compared to both Chow and HSD rats on most days.

**Figure 6:**
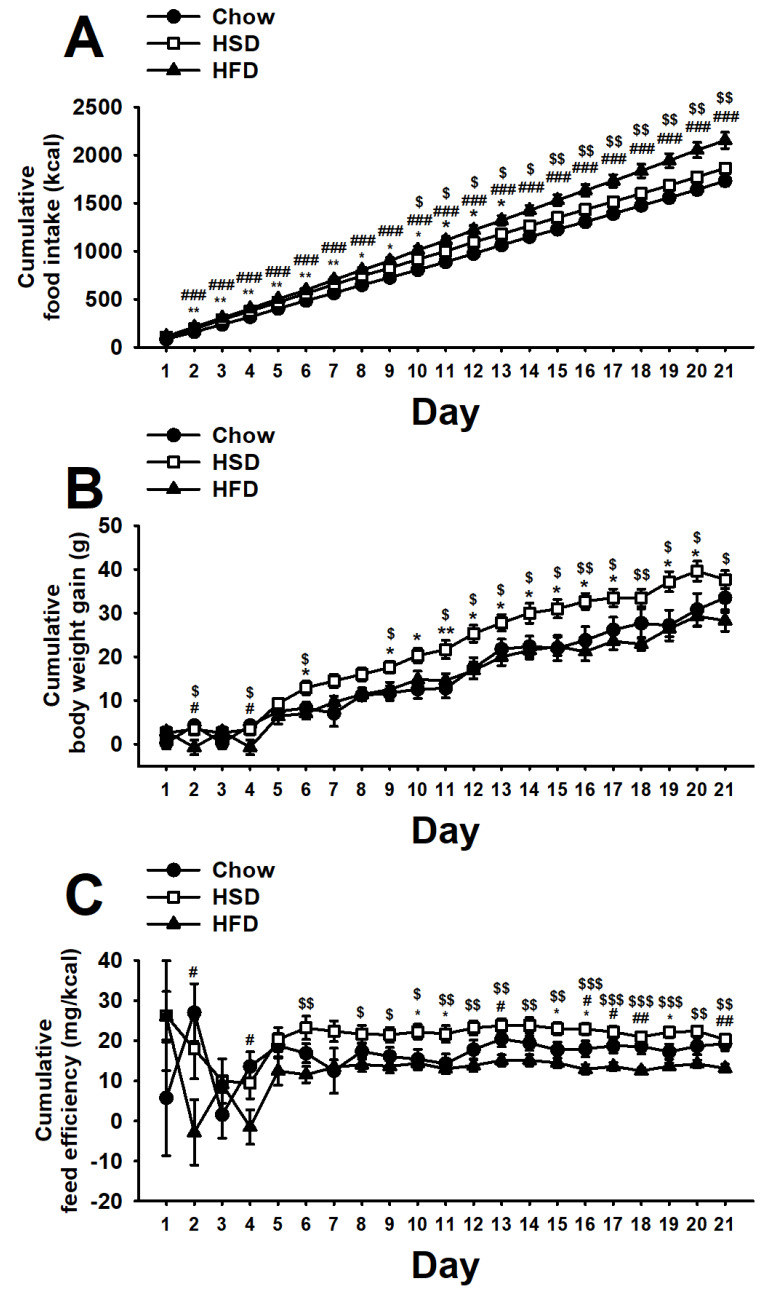
Effects of daily 1-h operant self-administration of Chow, HSD, or HFD on (**A**) cumulative food intake (kcal), (**B**) cumulative body weight gain (g), and (**C**) cumulative feed efficiency (mg/kcal) in female Sprague Dawley rats (*n* = 10/group). Rats were given the chance to self-administer one of the three diets in a fixed ratio 1 schedule during a 21-day period. Chow was otherwise freely available in the home cages. Panels represent *M* ± SEM. Symbols denote the following: * HSD differs from Chow, *p* < 0.05, ** *p* < 0.01; # HFD differs from Chow, *p* < 0.05, ## *p* < 0.01, ### *p* < 0.001; $ HSD differs from HFD, *p* < 0.05, $$ *p* < 0.01, $$$ *p* < 0.001 (Newman–Keuls test).

Therefore, the pattern of food intake of HFD rats in both the 1-h operant sessions and in the 23-h home-cage was such that it made this group consume cumulatively more energy than any other group throughout the study. Instead, HSD rats showed an initial increase in body weight gain as compared to controls, which then disappeared towards the middle of the study as the 23-h home cage hypophagia became more consistent (diet: F(2,27) = 11.46, *p* < 0.001; day: F(20,540) = 2442.21, *p* < 0.001; diet * day: F(40,540) = 10.81, *p* < 0.001; Figure 6A). Surprisingly, despite eating more calories than chow rats, HFD rats did not reliably show any difference in body weight gain compared to controls, while HSD rats showed a reliable increase in body weight gain as compared to the other groups throughout the study (diet: F(2,27) = 5.12, *p* < 0.05; day: F(20,540) = 210.84, *p* < 0.001; diet * day: F(40,540) = 2.93, *p* < 0.001; Figure 6B). Consistent with a high body weight gain and a low caloric intake, HSD rats showed a higher feed efficiency as compared to any other experimental group throughout the study (diet: F(2,27) = 4.17, *p* < 0.05; day: F(20,540) = 3.21, *p* < 0.001; diet * day: F(40,540) = 1.89, *p* < 0.01; Figure 6C). Conversely, HFD rats, which gained as much weight as Chow rats but consumed fewer calories, showed the smallest feed efficiency than any other group. Therefore, HSD rats consumed fewer kcal but gained more weight than HFD rats.

## 4. Discussion

In this study, we show that limiting access to either a high-sucrose or a high-fat diet induces binge-like eating. However, we found that the nature of the two binge-like eating behaviors is substantially dissimilar, and differences can be summarized as follows: (1) binge-like eating of the high-sucrose diet is based on an “active” increase of the amount food ingested, while binge-like eating of the high-fat diet is based on a “passive” increase in energy consumption, due to the high energy density of the diet; (2) binge-like eating of the high-sucrose diet is accompanied by an increased eating rate, while limiting access to the high-fat diet is not; (3) rats bingeing on the high-sucrose diet decrease the home-cage intake of the standard chow diet, while rats bingeing on the high-fat diet keep on consuming the same amount of standard chow upon return to the home-cage; (4) since the rats bingeing on the high-fat diet do not undereat standard chow in the home cage, they end up consuming cumulatively more energy than the rats bingeing on the high-sucrose diet; and (5) rats bingeing on the high-sucrose diet consume less energy, but gain more body weight and are, therefore, more energy-efficient than rats bingeing on the high-fat diet.

### 4.1. Binge-Like Eating Behavior Development

We found that female rats, when given the opportunity to self-administer either a high-sucrose or a high-fat diet, consume more energy than controls, as is typically observed in animal models of binge-like eating, independently from the diet offered. The 1-h food intake in both HSD and HFD groups increased in magnitude nearly two-fold compared to that of the Chow control rats. Binge episodes were significant in size as both HSD and HFD rats consumed between 35% and 40% of their daily intake in a single hour of exposure. The energy consumed (~40 kcal) by both HSD and HFD rats in this study was comparable to the energy consumed by rats in other similar limited access procedures to highly palatable food [4,7,8,10,30,31]. Therefore, based on the simple calculation of the energy consumed during the daily 1-h sessions, both diets were able to isomorphically model binge-like eating: (i) both HSD and HFD female rats consumed, in a small period of time, an amount of food which is generally not eaten under similar circumstances in normal conditions (i.e., Chow control group); (ii) binge-like eating occurred repeatedly over an extended period of time; and (iii) binge-like eating occurred in absence of hunger as rats were never experimentally food restricted/deprived [1].

However, the operant conditioning procedure allowed for a deeper analysis of the feeding behavior, revealing that binge-like eating in the two experimental groups was substantially different. While as previously shown and according to a learning process, the excessive energy intake of the high-sucrose diet develops slowly as a function of the days of limited access [12,13,14,15,16,17,18], rats with limited exposure to the high-fat diet consumed more calories than controls since the very first day of access. However, the higher energy consumption of the HFD group was exclusively due to the higher energy density of the food, unlike in the HSD group, whose 1-h self-administration intake was due to a true increase in the amount of food ingested. In other words, HFD rats consumed the same amount of food (grams) compared to controls, but, since it was more energy-dense than the standard chow diet, they ended up consuming more energy since the first day of access without any reliable escalation throughout the period of access. Therefore, we can confidently say that the rats exposed to the high-sucrose diet showed “active” binge eating, which developed as a function of the number of exposures, while rats exposed to the high-fat diet showed “passive” binge eating, which did not substantially change across the days of access.

Unexpectedly, our results revealed that rats bingeing on the high-sucrose diet or the high-fat diet showed important differences in both the rate and the regularity of food intake. As observed before in previous studies by our laboratory [12,13,14,15,16,17,18], binge-like eating of the high-sucrose diet is accompanied by a dramatic decrease in the inter food interval (i.e., an increase in eating rate, a hallmark of increased palatability). Conversely, the passive binge-like eating of the high-fat diet occurred with the same eating rate as controls. Therefore, we can confidently say that the high-fat diet was not more palatable than the standard control diet, likely contributing to the lack of increase in the amount of food ingested. This interpretation is also supported by another finding, which further emphasizes the differences between behavioral outcomes observed in the HSD and HFD rats: while rats exposed to the high-sucrose diet in the operant conditioning boxes showed a decrease in the intake of the standard chow diet upon return to the home-cage, rats exposed to the high-fat diet did not and kept eating as much as the controls. This finding is particularly relevant, because it may also help to clarify the nature of the hypophagia of a less preferred food alternative in more general terms. Undereating of less preferred food has been traditionally explained as a corrective energy-homeostatic response to recent excessive energy intake [32,33]. However, other explanations that do not involve energy-homeostatic mechanisms have been hypothesized for this phenomenon, having instead to do with non-nutritional and hedonic components of feeding. These alternative interpretations include a “negative contrast” [4,21,34], due to the close temporal proximity to a more rewarding alternative [35,36], and “withdrawal” from palatable food, similar to the negative emotional state observed with drugs of abuse [34,37]. Our findings strongly support the notion of a non-homeostatic, hedonic mechanism underlying the hypophagia of the less preferred chow diet in the home-cage. Using an argumentum ad absurdum, if the hypophagia were simply due to a corrective energy-homeostatic response to the previous caloric load, then we should have observed the hypophagia of the standard chow diet in the home-cage in both the HSD and the HFD groups. This was instead not the case, as only rats exposed to the high-sucrose diet (but not those exposed to the high-fat diet), which showed an “active” increase in the amount of food eaten and an increased eating rate due to the palatability of the diet, showed the hypophagia of the standard chow diet in the home-cage. In support of this interpretation, we also found that the more, the faster, and the more regularly HSD rats ate during the 1-h binge-like eating sessions, the less food they were consuming in the home-cages. Such correlations were found in neither chow nor HFD rats. Therefore, we can reasonably speculate that the high-fat diet was not more palatable than the standard chow diet, as was the case for the high-sucrose diet. For this reason, female rats exposed to the high-fat diet did not ingest a larger amount of food than controls in the operant boxes, did not eat faster than controls, and did not reject the home cage standard chow as high-sucrose diet rats did. Future studies directly comparing the three diets in a progressive ratio schedule of reinforcement may help us to understand the contribution of reinforcing efficacy on the outcomes observed in this paper.

It is important to emphasize that other widely used animal models of binge-like eating based on limited access to a high-fat diet have shown home-cage hypophagia [7] and that, although the diets of both studies are highly saturated fat based, the different behavioral outcomes observed may still be due to differences in fat composition. Consistent with this potential explanation, it has been shown that oils with different carbon chain lengths, unsaturated states of the fatty acid, and carboxyl groups can be differently palatable as measured by licking rate [26]. Future studies will be important to make direct comparisons among diets with different fat sources using the operant binge-like eating animal model.

### 4.2. Cumulative Intake, Body Weight Gain, and Feed Efficiency

In this study we observed that the rats exposed to the high-fat diet consumed more energy than both control and HSD rats. This outcome resulted from both the passive intake in the 1-h self-administration procedure and the inability to reduce the intake in the home cage. Surprisingly, rats exposed to the high-fat diet, despite consuming more energy than any other group, did not gain more weight than the controls. Instead, rats exposed to the high-sucrose diet, which consumed less energy than HFD, were the heaviest. Therefore, HSD rats consumed fewer calories but gained more weight than HFD rats, resulting in a more energy-efficient phenotype. These results are consistent with other animal models of binge-like eating. While limiting access to high-fat diets generally does not affect body weight gain [7,30], conversely, several limited access procedures to sucrose diets have been shown to increase body weight gain [4,31,38]. Future studies will be needed to determine the mechanism underlying the different metabolic effects of limited access to the two diets.

Translating the results of this study to individuals affected by BED is not trivial. The investigation of how diet composition affects binge eating in humans has often produced conflicting findings [39,40,41]. In addition, while the composition of the diet in animal studies is experimentally determined and, therefore, easy to achieve, this represents a more difficult task to achieve in humans and, given the richness of human diets, results are more difficult to interpret.

## 5. Conclusions

In this study we show that limiting access to either a high-sucrose or a high-fat diet can produce very different outcomes. Although both of the diets, when provided for only one hour per day, can promote binge-like eating, the nature of the two excessive behaviors is substantially different; binge-like eating of the high-sucrose diet is based on a true increase in the amount of food consumed and an increase in eating rate, suggesting increased palatability, and a decrease in the home-cage standard chow intake. Conversely, binge-like eating of the high-fat diet is a result of passive energy consumption and the high energy-density of the food. Additionally, high-fat diet binge-like eating is accompanied by neither an increased eating rate nor the rejection of the standard chow diet in the home-cages. Moreover, while the high-sucrose diet bingeing rats consume less energy than high-fat diet bingeing rats, the former are more energy efficient and gain more body weight than the latter. These results provide information on how the quality of the food can deeply influence the behavioral and metabolic outcomes of binge-like eating.

## Supplementary Materials

The following are available online at https://www.mdpi.com/2072-6643/12/6/1610/s1, Table S1: Diets, Table S2: Composition of custom Bio-Serv F07679 diet.
