# Supplementary material for: Consummatory, Feeding Microstructural, and Metabolic Effects Induced by Limiting Access to Either a High-Sucrose or a High-Fat Diet
Source: Nutrients. 2020 May 30;12(6):1610. doi: 10.3390/nu12061610 (PMC7352440; doi:10.3390/nu12061610)
Supplement: Supplementary file 1 [file nutrients-12-01610-s001.pdf]

nutrients

MDPI

Article

Consummatory, feeding microstructural and metabolic effects induced by limiting access to either a high-sucrose or a high-fat diet

Harrison Lee [1], Elisa Giunti [1], Valentina Sabino [1] and Pietro Cottone [1,*]

Laboratory of Addictive Disorders, Departments of Pharmacology and Psychiatry, Boston University School of Medicine, Boston, MA, USA
* Correspondence: cottone@bu.edu; 001-617-358-1950

Supplementary Table S1. Diets

		Diet		
		Chow	HSD	HFD
Manufacturer				
		TestDiet	TestDiet	Bio-Serv
Diet ID				
		5TUM	5TUL	F07679
Macronutrient Composition (kcal %)				
	Protein	24.1	20.6	15.0
	Carbohydrate	65.5	66.7	25.0
	Fat	10.4	12.7	60.0
Fiber (g %)				
		4.4	4.9	4.8
Energy density (kcal/g)				
		3.30	3.44	5.23

Supplementary Table S2. Composition of custom Bio-Serv F07679 diet

Ingredient	g/kg
Casein	210.0
L-Cysteine	3
Corn Starch	0.0
Dextrose	222
Sucrose	100.0
Hydrogenated Cottonseed Oil	325.0
Soybean Oil	25.0
Cellulose	50
Mineral Mix (AIN-93G)	35.00
Mineral Mix (AIN-93)	10
Choline Bitartrate	2.5
tBHQ	0.0014

Tableting Aids	17.5
Total	1000
